# Two Cases of Sloughing Ulceration, in Which Repeated Bleeding Was Employed

**Published:** 1826-08

**Authors:** 

**Affiliations:** the St. James's Infirmary


*Two Cases of Sloughing Ulceration, in which repeated Bleeding was employed.* Treated at the St. James's Infirmary, by Mr. Rose.

Case I. *Sloughing Sore of the Penis.*

David Andrews, thin, and of a dark complexion, twenty-three years of age, was admitted into the St. James's Infirmary, on the 27th April, 1817, under the care of Mr. Rose. He had a deep sloughing sore on the inner prepuce, immediately behind the corona glandis, which had burrowed into and destroyed the greater part of the glans, almost entirely separating that body from the extremities of the corpora cavernosa: it had also penetrated into the urethra, allowing the urine to pass through the wound. The whole of the sore was covered with a deep slough; the edges were jagged, and surrounded by a dark red margin. His tongue was dry and rough. He was emaciated; had much thirst, with a flushed countenance, a hot skin, and a quick small pulse. He complained of burning pain in the sore, which he said had been somewhat relieved by a bleeding that took place from it on the preceding day. The sore had been present for a fortnight, and he had taken mercurial pills from its first appearance.

Fiat V.S. ad ℥xij.—℞. Extracti Opii, Ext. Conii, āā ℈i.; Aquæ font. ℥ iij. M. fiat lotio diligenter utenda; et applicetur Lotio Saturn. circa penem. —℞. Magnesiæ Sulphatis, ʒiss.; Liquor Antimon. Tartariz. m. xxv.; Aquæ Menth. Pip. ℥ iss. M. quarta quaque hora sumend.—Capiat Pulv. Ipecac. Comp. gr. xv. hora somni.—Diet, Tea and Barley-water.

28th.—The sloughing goes on. The blood is very much cupped. Bowels open; pulse sharp and quick.

Repet. V.S. ad ℥ xiv. et repet. cætera.

29th.—Continues very feverish. Pulse 100; little or no sleep; skin dry and hot; tongue furred; lips parched. The blood taken yesterday cupped and buffy. The sloughing has extended itself in the glans, the greater part of which is gone. The dark mortified part terminates abruptly in the shining red margin, and there is no appearance of separation.

Repet. V.S. ad ℥ xij. Repet. med. et lotio.

30th.—The blood taken yesterday is but slightly cupped or buffy. He slept a little, and is less irritable. The sore is quieter.

Pergat, et adhibeatur Cataplasma commune vespere.

May 1st.—Some healthy pus appears in the wound, which is easier.

Repet. Catap. et cætera.—Beef-tea frequently.

4th.—The sore has rapidly improved: the sloughs are separated, and granulations every where appear. Has left off his medicine since yesterday.

To have nourishing diet, and take an infusion of bitters with Epsom salts.

8th.—Wound dressed with Bates's camphorated lotion, and healing favourably.

Porter, &c. Omit medicines.

27th.—Sore entirely healed.

CASE II. *Sloughing Sore of the Genitals in a Female.*

Mary Gardiner, nineteen years of age, thin and pale complexioned, applied as an out-patient at the St. James's Infirmary, on the 11th of August, 1821, on account of two extensive sores, of a foul and sloughy appearance, with excoriating discharge, situated within the edge of the vestibulum; one at the upper part of the left labium pudendi, and the other on the same labium nearer the perineum. She stated that she had been ill about two months.

Mr. Rose directed her to use a simple lotion, to take some aperient medicine, and to return next day; but she ceased attending at the Infirmary, and was not seen again until the 16th of September, when she was visited at her own house. She was then much emaciated, and unable to leave her bed. She stated that she had been rubbing-in mercurial ointment, night and morning, from the middle of August until the 8th or 9th of September. About the 24th of August, she was profusely salivated; and at that time the sores became more painful, and begun to spread. She left off the ointment for a few days, but returned to it again, and continued it for the first eight or nine days of September. Her gums on the 16th were still much ulcerated. The two sores on the genitals had run into one, and had destroyed a considerable part of the left labium and nympha, extending some way towards the pubis and groin. The base of this sore was covered with a dark ash-coloured slough, the margin was jagged and irregular. Towards the groin, a segment of skin, of about an eighth of an inch in width, overlapped the ulcer; which segment was completely sphacelated and black. The integuments adjoining this were of a deep shining red colour. There was no appearance of suppuration, but a good deal of excoriating ichorous discharge. She complained of violent burning pain; had a quick, small pulse, about 130; a flushed face, a dry furred tongue, and a very hot skin. Her bowels were open; she had no appetite, but constant thirst. Had been taking wine and spirits to support her strength.

Fiat V.S. ad ℥ xiv.—℞. Magnes. Sulphat. ʒi.; Liq. Antimon. Tart. ʒss.; Aquæ Menth. Pip. ʒxiss. M. fiat haustus quarta quaque hora sumendus.—Adhib. Aqua fontana diligenter ulceri.

September 17th.—The blood is buffy and slightly cupped. There is no material change in her symptoms. The sore has rather increased, but she thinks the cold application gives her ease; and the thirst is not so incessant. Pulse as yesterday; several stools.

Repet. V. S. ad ℥ x.—Repet. medicamenta.—To have a cupful of weak beef-tea occasionally.—Diluents freely.

18th.—The sore has considerably increased, but the surrounding redness has diminished, and a little good pus oozes out amongst the sloughs. She says the pain is much relieved, except when the urine passes. Her tongue is more moist; her pulse 120, soft and feeble; her skin is more natural. Slept a little in the night. The blood taken yesterday is buffy, but not cupped. Several stools.

> She is directed to continue the cold application, and to use a little soft ointment on lint, to defend the parts, before she empties the bladder.—To have beef-tea often, and a little Cape wine.—℞. Mist. Camphoræ, ℥vj.; Ammon Subcarb. gr. xij. M. capiat ℥iss. quater die.—℞. Pulv. Ipecac. Comp. gr. viij. hora somni sumendus.—Omitt. haustus.

19th.—The sloughs, in one or two parts, are beginning to separate, and some spongy granulations appear. She slept well. Pulse 112.

> Adhib. Catap. ex farina Sem. Lini.—℞. Mist. Camphoræ, ℥ij.; Decoc. Cinchonæ, ℥iv.; Ammoniæ Subcarb. gr. xij. Capiat ℥iss. quater die.—Good broth often.

21st.—Sloughs all detached round the edges, and beginning to separate from every part of the sore.

> ℞. Decoct. Cinch. ʒxj.; Tincturæ Ejusd. ʒi.; Acid. Sulph. dil. m. x. M. quarta quaque hora sumend.

25th.—The sloughs are all separated; and the wound lightly dressed with lint, covered with a pledget of simple ointment.

October 1st.—Wound healing favourably; strength gradually improving; bowels relaxed.

> To apply a flannel bandage.—Wound dressed with Bates's camphorated lotion.—Omitt. med.—Capiat Mist. Cret. c. Tinct. Opii. p. r. n.

3d.—Bowels quiet since the 1st.

> Capiat Decoct. Sarsæ. Oj. quotidie.

The sore was not entirely healed until the 25th of November, owing to her extreme debility.

---

